# A Case of Preoperative Diagnosis of Pulmonary Artery Aneurysm Resected by Segmentectomy

**DOI:** 10.7759/cureus.56984

**Published:** 2024-03-26

**Authors:** Jun Amioka, Yoshinori Handa, Tatsuya Katayama

**Affiliations:** 1 Thoracic Surgery, Hiroshima Prefectural Hospital, Hiroshima, JPN

**Keywords:** segmentectomy, pulmonary artery, minimally invasive surgical procedures, lung, hemorrhage, aneurysm

## Abstract

A pulmonary artery aneurysm (PAA) is a rare condition. It is treated in various ways, depending on its location and size. Herein, we describe the preoperative diagnosis of a PAA that was resected by segmentectomy. A 44-year-old female underwent CT, which revealed a 15-mm saccular protrusion in the right pulmonary artery and was diagnosed with PAA. The patient was initially observed without requiring further treatment, but a gradual increase in size led to therapeutic intervention. Because the PAA was located just peripheral to the right A8 bifurcation, embolization using interventional radiology was deemed too difficult. Therefore, a surgical intervention was planned. Subsequently, S8 segmentectomy, basal segmentectomy, and basilar pulmonary artery ligation were all considered. Ultimately, basal segmentectomy was selected because it allowed the resection of the pulmonary artery and did not result in invalid ventilation of the basal segment. A basal segmentectomy was performed, and the PAA was safely removed without hemorrhage. Histopathological examination revealed arterial and venous wall-like areas, and the patient was diagnosed with pulmonary artery malformation. A PAA is typically treated with coil embolization, ligation of the pulmonary artery, aneurysmectomy, and lung resection; however, no clear treatment guidelines exist. After discussion, we selected basal segmentectomy as a safe and minimally invasive procedure, and we resected the PAA without complications. The optimal treatment strategy for PAAs varies according to location and size, and a careful treatment plan should be established.

## Introduction

Pulmonary artery aneurysm (PAA) is a rare disease wherein the pulmonary artery wall becomes weakened and locally dilated owing to organic changes. According to the pathological autopsy of Deterling and Clagett, PAA is only reported in eight out of 109,571 cases [1]. Most PAAs are asymptomatic and are frequently discovered incidentally [2]. However, PAAs are potentially fatal diseases with a risk of pulmonary artery dissection and pulmonary aneurysm rupture [3,4]. The treatment of PAA involves coil embolization, ligation of the pulmonary artery, aneurysmectomy, and lung resection. This report details the case of a patient with a PAA wherein segmentectomy was selected to resect the PAA with minimal pulmonary resection and to avoid disabling ventilation, which ultimately demonstrated favorable results.

## Case presentation

**Figure 1:**
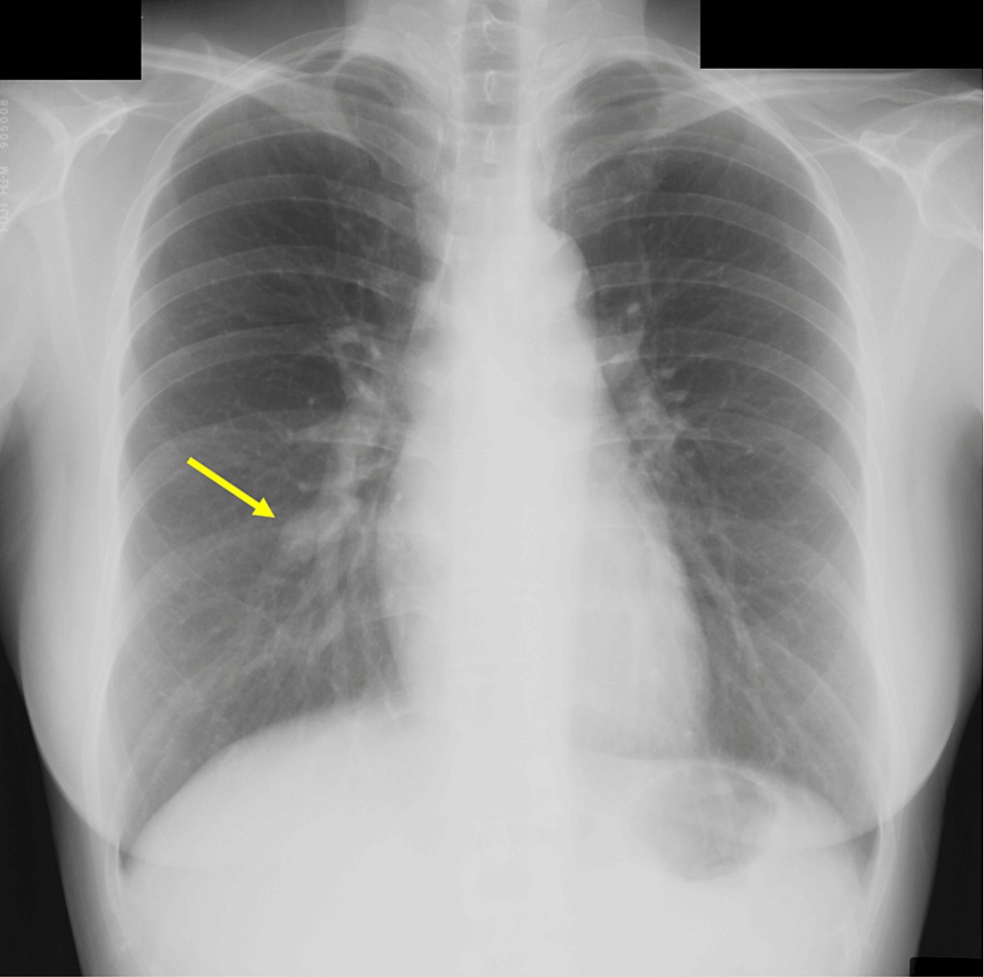
Preoperative imaging findings Chest radiograph shows the nodule in the right lower lung field (yellow arrow).

A 44-year-old female was referred to our hospital after an abnormal chest shadow was noted during a checkup. The patient had no history of smoking but had an anterior chest burn at the age of one and a history of skin graft treatment at the age of five. Blood tests revealed no significant abnormalities. Chest radiography revealed a 15-mm nodular shadow of a blood vessel in the right lower lung field (Figure 1).

**Figure 2:**
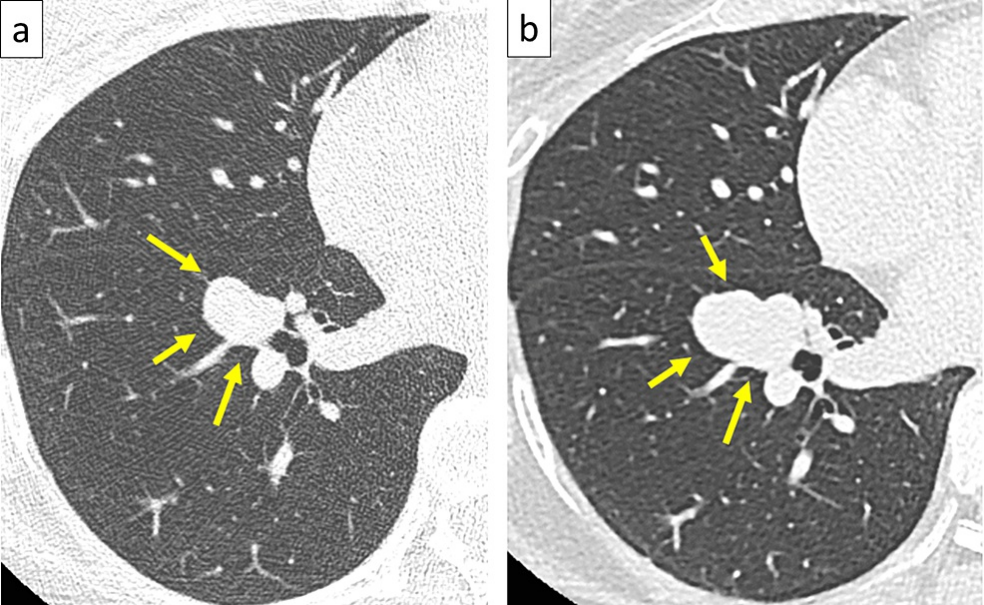
Preoperative imaging findings (a) CT image showing a 15-mm saccular protrusion in the A8 bifurcation of the right pulmonary artery at the time of the initial visit (yellow arrows). (b) Seven years later, a CT image shows the PAA enlarged to 26 mm. CT: computed tomography

A chest CT showed a 15-mm saccular protrusion at the A8 bifurcation of the right pulmonary artery. No obvious outflow vessels were observed, and PAA was diagnosed (Figure 2a).

Owing to its small size and the patient’s wishes, further intervention was not planned. However, the size of the pulmonary aneurysm gradually increased. Seven years after the diagnosis, the aneurysm was 26 mm. Considering the risk of rupture, the decision was made to intervene with further treatment (Figure 2b and Video 1).

**Video 1:** Preoperative imaging findings of 3D CT CT: computed tomography; 3D: three-dimensional

**Figure 3:**
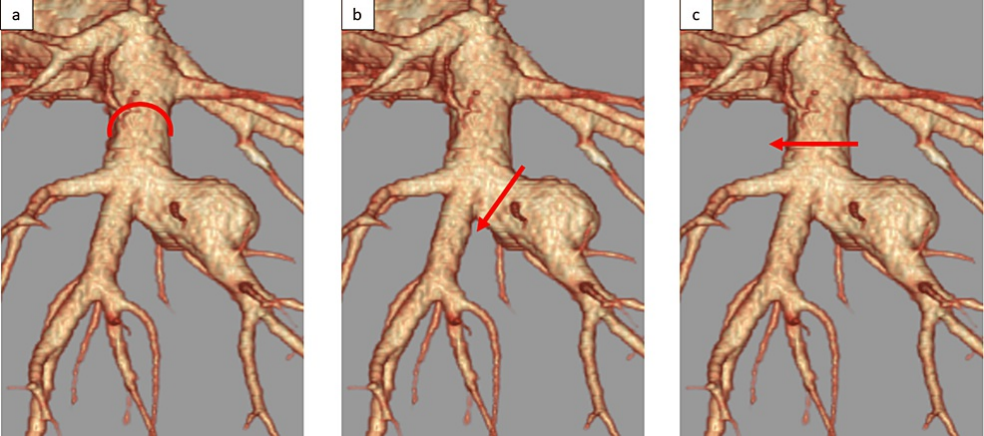
Preoperative imaging findings (a) pulmonary ligation; (b) S8 segmentectomy; (c) basal segmentectomy

Potential treatments included coil embolization, ligation of the right basilar pulmonary artery, S8 segmentectomy, and basal segmentectomy. Coil embolization was not considered an acceptable choice because the pulmonary aneurysm was located at the A8 bifurcation, which would have embolized other basilar pulmonary arteries. Ligation of the right basilar pulmonary artery was not acceptable because it would have resulted in invalid ventilation of the right basilar segment (Figure 3a).

S8 segmentectomy and aneurysmectomy were not acceptable because of the risk of intraoperative and postoperative bleeding since the vessels would have to be resected in the vulnerable PAA (Figure 3b). Finally, right basal segmentectomy was selected because it is a safe vascular resection and does not result in invalid ventilation (Figure 3c). A right basal segmentectomy was performed using video-assisted thoracoscopy (Video 2).

**Video 2:** Intraoperative movie showing the PAA was beating and appeared very soft and fragile PAA: pulmonary artery aneurysm

**Figure 4:**
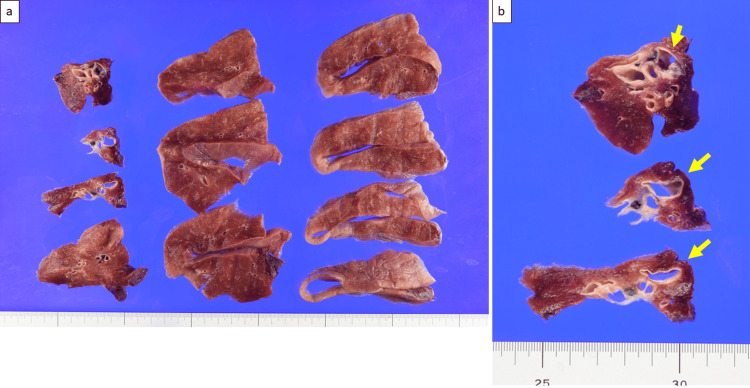
Macroscopic findings of AVM (a-b) The AVM is saccularly dilated (yellow arrows). AVM: arteriovenous malformation

PAA was observed between the middle and lower lobes, showing pulsation and appearing soft and fragile. A8 was not identified, and the basilar artery was resected. Subsequently, the basilar vein and basilar bronchus were resected. Finally, the intersegments were separated, and a right basal segmentectomy was performed. The surgical duration was 123 minutes, and there was 25 mL of blood loss. No postoperative hemorrhage or air leaks were observed, and the drain was removed the day after surgery. The patient was discharged on postoperative day six. Macroscopic findings showed that A8 was saccularly dilated (Figure 4a-4b).

**Figure 5:**
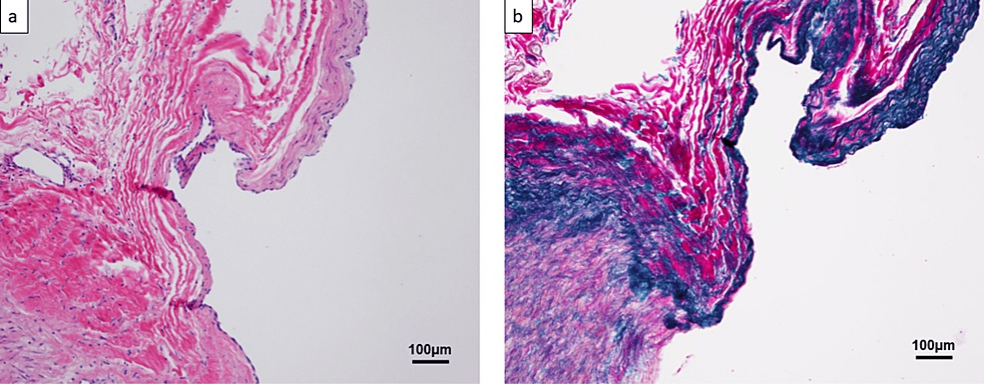
Histopathological findings of AVM (a) The vessel wall is nidus and is indistinguishable from arteries or veins. (b) Verhoeff-van Gieson staining shows arterial and venous wall-like areas of the vessel wall. AVM: arteriovenous malformation

Histopathological analysis revealed that the vessel wall was a nidus indistinguishable from the arteries or veins (Figure 5a).

Verhoeff-van Gieson staining showed arterial and venous wall-like areas of the vessel wall (Figure 5b). Irregular thickening of the intima with partial mucous seed-like degeneration was also observed. The diagnosis was pulmonary arteriovenous malformation (AVM) and not PAA. Six months after surgery, a follow-up CT scan showed that the pulmonary aneurysm was completely resected. The patient had no respiratory distress symptoms and had a good course.

## Discussion

PAA is a rare condition with no treatment guidelines. It carries the risk of rupture and pulmonary artery dissection and requires an appropriately timed therapeutic intervention. Various treatment methods for PAA exist, depending on the location and size of the lesion. In this case, the PAA was located at the S8 bifurcation, and a basal segmentectomy was selected after careful consideration. Consequently, we encountered a case in which the PAA was resected with minimal lung resection and a good course was achieved without impairing ventilation.

Two types of pulmonary aneurysms exist: true PAAs and pseudoaneurysms. PAA is a localized dilation of all three layers of the vessel wall, whereas a pseudoaneurysm is a dilation of any of the vessel wall layers. Pseudoaneurysms are at a greater risk of rupture, and aggressive treatment interventions are important. PAA has congenital and acquired etiologies. A review of the literature by Gupta et al. found that 63 (25%) of 248 patients had congenital PAA [5]. Congenital etiologies include congenital heart disease, such as Eisenmenger's syndrome; pulmonary valvular abnormalities, such as pulmonary artery stenosis; and connective tissue abnormalities, such as Marfan syndrome. In contrast, acquired causes include pulmonary hypertension, autoimmune disease, vasculitis, and idiopathic for true pulmonary aneurysms, while trauma, tuberculosis or syphilis infection, and malignancy are causes for pseudoaneurysms. The patient had no congenital disease or history of pulmonary hypertension and was preoperatively diagnosed with idiopathic PAA.

The normal upper limits of the main pulmonary artery on CT are 29 mm in men and 25 mm in women [6]. PAA is generally considered a dilation exceeding 1.5-fold above the normal range [5]. They are also classified as proximal or distal PAAs, depending on their location. Proximal PAAs refer to aneurysms involving large pulmonary arteries, such as the main pulmonary artery. Conversely, distal PAAs are aneurysms involving the distal arteries [7]. This case involved a 15 mm saccular protruding pulmonary artery located in the S8 bifurcation. Although no clear criteria exist for diagnosis, it was larger than a normal pulmonary artery and was preoperatively diagnosed as a PAA. The aneurysm was classified as a distal PAA because it was located at the S8 bifurcation.

PAA rarely causes symptoms and is often discovered incidentally. Similarly, no symptoms were observed in our patient, and the disease was discovered during a check-up. However, an enlarged pulmonary aneurysm in some patients may compress surrounding organs such as the trachea or coronary artery, or rupture and dissection may occur, resulting in symptoms such as shortness of breath, bloody phlegm, chest pain, palpitations, and episodes of syncope [2].

**Figure 6:**
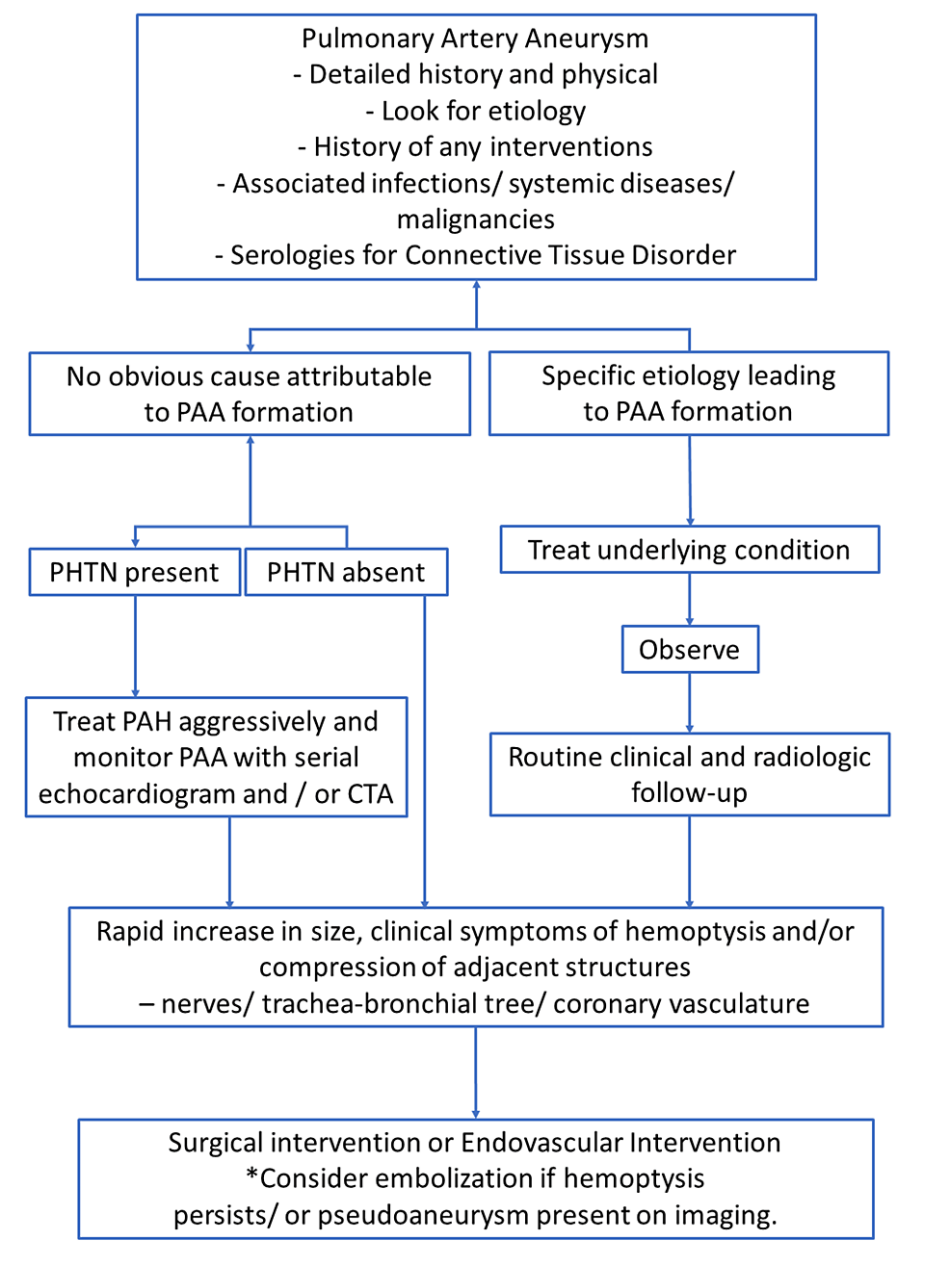
Management algorithm for a PAA PAH: pulmonary arterial hypertension; PAA: pulmonary artery aneurysm; PHTN: pulmonary hypertension; CTA: computed tomography angiography * indicates a special point to be considered. Copyright/license: This figure has been adopted from Gupta et al. [5], which is an open-source article distributed under the terms and conditions of the Creative Commons Attribution-NonCommercial 4.0 License (https://creativecommons.org/licenses/by-nc/4.0/).

The causes of PAA vary, and treatment strategies must be determined based on the causative disease, comorbidities, and hemodynamics. Conservative treatment includes medical management of the causative disease or comorbidity and management of pulmonary hypertension. Gupta et al. provided an algorithm for the management of PAA in a literature review (Figure 6) [5].

Graft interposition, pulmonary artery plication, lobectomy, and bilobectomy are surgeries often performed for proximal PAAs [5,8]. There is a recent report on endovascular treatment of the proximal pulmonary artery [9]. In contrast, coil embolization, pulmonary artery ligation, pulmonary aneurysmectomy, lobectomy, segmentectomy, bilobectomy, wedge resection, and stenting are acceptable options for distal pulmonary aneurysms. Our treatment strategy for this distal PAA was initially considered a less-invasive endovascular intervention. Wedge resection, pulmonary aneurysmectomy, or pulmonary artery ligation were subsequently considered. Anatomic resection (segmentectomy, lobectomy, bilobectomy) that minimizes the amount of lung resection should be considered if the PAA is centrally located and too much invalid ventilation from pulmonary artery ligation is observed, or if wedge resection or pulmonary aneurysmectomy is difficult. Although basal segmentectomy was acceptable in this case, S8 segmentectomy was also considered, with the right main pulmonary artery and inferior pulmonary vein secured. We performed basal segmentectomy (which is a safer resection owing to the risk of bleeding) based on the patient’s desire for a safe procedure.

A typical CT finding in PAA is saccular dilatation of the pulmonary artery. In contrast, the typical CT findings of AVM are round to oval nodules with inflow and outflow vessels. A PAA can cause dissection or rupture, whereas an AVM can cause cerebral infarction, brain abscess, hypoxemia, or rupture [10-12]. The treatment varies from case to case; however, endovascular intervention or surgical resection is the mainstay for both PAA and AVM. In a true PAA, a three-layered structure of enlarged saccular arteries is present, whereas a tear in the tunica media occurs in a pseudoaneurysm. In contrast, AVMs show irregular vessel wall construction and a mixture of arterial and venous areas. This patient was diagnosed with PAA based on preoperative CT findings, although the pathological results showed an AVM. Even if a preoperative diagnosis of AVM had been made, the patient would have been at risk of cerebral infarction and would have still required therapeutic intervention. Furthermore, the location and size of the lesion would have required a basal segmentectomy.

## Conclusions

Appropriate management, timing, and methods of therapeutic intervention are important in each case of PAA. Endovascular intervention, pulmonary artery ligation, lung resection, and PAA resection may be considered potential surgical treatments. However, further cases are required to establish a standard of care.
